# Opportunities and challenges of hepatocellular carcinoma organoids for targeted drugs sensitivity screening

**DOI:** 10.3389/fonc.2022.1105454

**Published:** 2023-01-06

**Authors:** Cuiying Xie, Ancheng Gu, Muhammad Khan, Xiangcao Yao, Leping Chen, Jiali He, Fumiao Yuan, Ping Wang, Yufan Yang, Yerong Wei, Fang Tang, Hualong Su, Jiamin Chen, Jinxia Li, Bohong Cen, Zhongyuan Xu

**Affiliations:** ^1^ Clinical Pharmacy Center, Nanfang Hospital, Southern Medical University, Guangzhou, China; ^2^ National Medical Products Administration (NMPA) Key Laboratory for Research and Evaluation of Drug Metabolism and Guangdong Provincial Key Laboratory of New Drug Screening, School of Pharmaceutical Sciences, Southern Medical University, Guangzhou, China; ^3^ Department of Radiation Oncology, Affiliated Cancer Hospital and Institute of Guangzhou Medical University, Guangzhou, Guangdong, China

**Keywords:** organoids, targeted drugs, hepatocellular carcinoma, drug screening, tumor microenvironment

## Abstract

Hepatocellular carcinoma is one of the malignancies worldwide with a high mortality rate and an increasing incidence. Molecular Targeted agents are its common first-line treatment. Organoid technology, as a cutting-edge technology, is gradually being applied in the development of therapeutic oncology. Organoid models can be used to perform sensitivity screening of targeted drugs to facilitate the development of innovative therapeutic agents for the treatment of hepatocellular carcinoma. The purpose of this review is to provide an overview of the opportunities and challenges of hepatocellular carcinoma organoids in targeted drug sensitivity testing as well as a future outlook.

## 1 Introduction

Liver cancer is one of the fatal malignancies that remains a global health challenge. According to the GLOBOCAN 2020 estimates of cancer incidence and mortality produced by the International Agency for Research on Cancer, liver cancer ranks sixth in global cancer incidence, third in mortality, and has a low five-year overall survival rate (1, 2). Hepatocellular carcinoma (HCC) accounts for more than 90% of the primary liver cancer (PLC) (3). In addition, PLC includes cholangiocarcinoma (CC), mixed hepatocellular carcinoma (HCC/CC), and other types of liver cancer. The majority of HCC patients are diagnosed at an advanced stage, and currently, the multikinase inhibitors sorafenib and lenvatinib are the first-line molecularly targeted drugs approved by the FDA for the treatment of advanced HCC (4). Additionally, other molecular targeted drugs currently in clinical use include regorafenib, cabozantinib, and ramucirumab. More detailed molecular targeted drug therapy for HCC is fully described in the review *Molecular therapies and precision medicine for hepatocellular carcinoma* (5). However, the efficacy of clinical drug therapy is nevertheless constrained by tumor drug resistance and patient heterogeneity in response to drug therapy. There are still obstacles in the way of research on liver cancer treatments. Over the years, 2D models and Patient-Derived Xenograft (PDX) have been regarded as the standard models for the study of liver cancer (6, 7). However, these models fail to recapitulate critical growing features of tumors *in vivo*. Therefore, a novel stable model that can simulate the characteristics of HCC *in vitro* and accurately reproduce the drug response for drug testing and therapy regimen proposals for HCC is urgently needed.

Organoids were described as a three-dimensional structure produced from (pluripotent) stem cells, progenitor, and/or differentiated cells that self-organize through cell-cell and cell-matrix interactions to simulate some elements of the native tissue architecture and function *in vitro* (8). Since Sato et al.’s first use of single Lgr5 stem cells to generate crypt-villus structures in 2009 (9), the organoid technology has progressed considerably (10). And in 2015, the adult liver organoid model capable of long-term stable expansion *in vitro* was established. The need for disease research has led to the development of organoid models of liver cancer to study the initiation and progression of liver cancer with progressive applications in cancer development mechanisms (11–16), preclinical research (17), personalized medicine (18–21) and drug screening (22, 23). Many factors can induce the oncogenesis of the liver cancer, organoid can also be help to study the mechanism of oncogenesis and development of HCC. More detail can be found in the review *Novel patient-derived preclinical models of liver cancer* (17). And organoid models can be created and used to advance cancer treatment research for the benefit of patients. HCC organoids, which are often created from the tumor tissue of HCC patients, replicate tumor properties more accurately *in vitro* than other models, making them a suitable experimental model for a range of oncology research topics, including the sensitivity testing of targeted medications. The HCC organoid has several advantages over earlier designs, but it also has a number of shortcomings that need to be resolved.

This review focuses on the advantages and disadvantages of using HCC organoids as a reliable *in vitro* model for drug sensitivity testing in targeted therapeutic applications and the future outlook of the development of HCC organoids.

## 2 Establishment of HCC organoids

**Figure 1:**
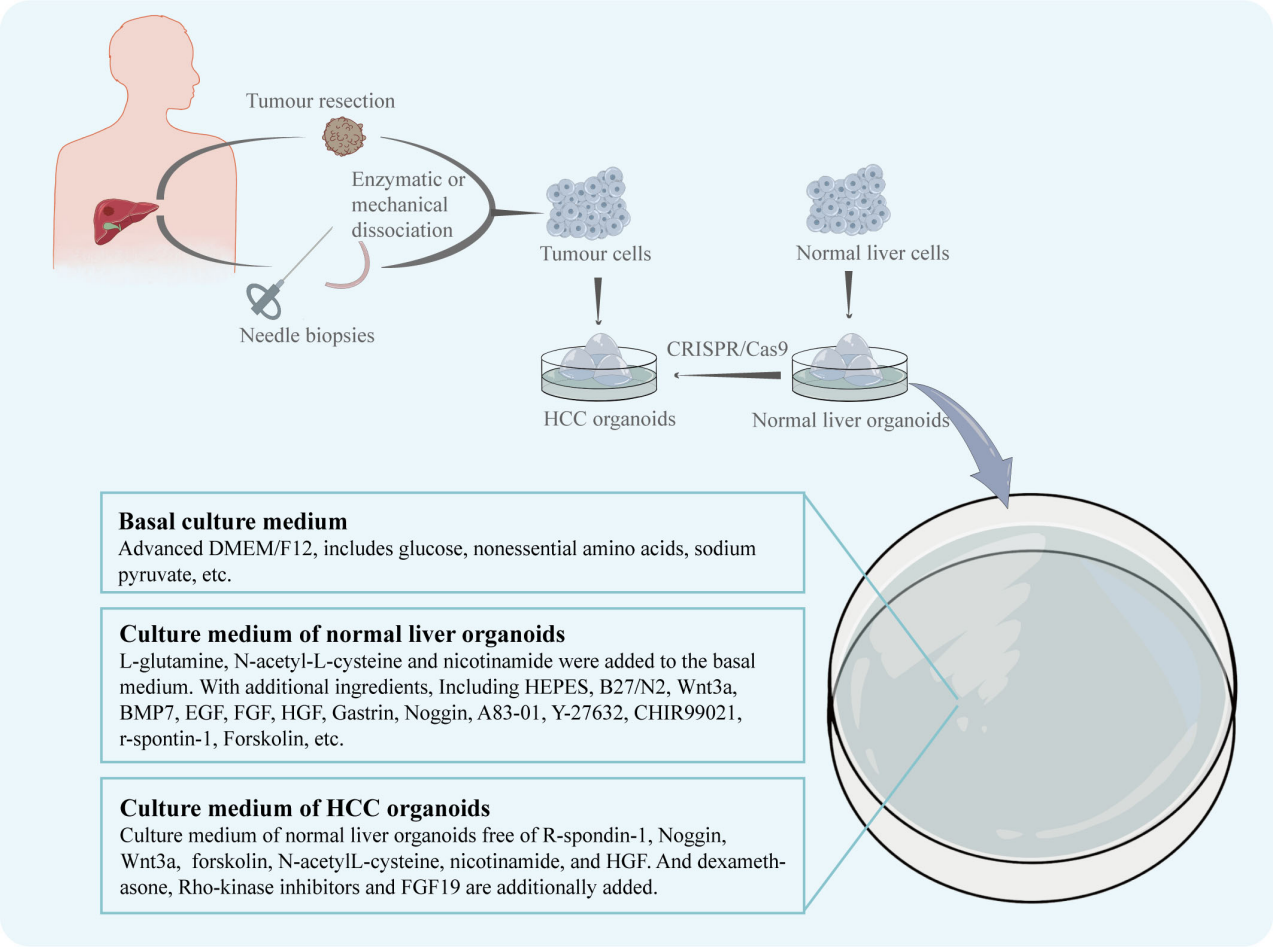
The patient's HCC tissue, obtained by intraoperative resection or needle biopsy, is enzymatically or mechanically broken down to obtain dispersed tumour cells. The tumour cells are cultured in dishes to obtain the HCC organoids. Normal hepatocytes from other sources are cultured in dishes to obtain normal liver organoids. The CRISPR/Cas9 system can be used to induce the generation of HCC organoids. The nutrient solution used for culture was adjusted according to the growth characteristics of HCC.

According to Marsee et al., in addition to developing organoids from stem cells, differentiated cells can also be used in the construction of organoids (8). And an organoid should have more than one type of the simulating organ’s cells and be capable of showing the structure and functionality specific to that organ (24). The CRISPR/Cas9 system can be used to introduce mutant genes into normal organoids to cause tumorigenesis, which is useful for the creation of various liver cancer organoids (25). Similar to this effect, oncogene overexpression may be induced in healthy liver organoids to produce HCC organoids. For instance, Sun et al. created an organoid that resembles HCC by making hiHep organoids overexpress the oncogene c-Myc, which is essential for the development of HCC (15). However, because the mechanism of the disease is still poorly understood, tissue from HCC patients is increasingly frequently used in the creation of HCC organoid models. The first HCC patient-derived organoid model was produced in 2017 by Broutier et al. (18) using tissue specimens collected from the intraoperative resections of eight HCC patients. The patient’s HCC tissues were divided using digestive solution and cultured in isolation medium devoid of Noggin, Rspo-1, and Wnt3a but with 3nM dexamethasone in order to produce organoids. The HCC organoid model they created duplicated the morphological characteristics as well as the genetic and transcriptome characteristics of the original tumor. Additionally, human tissue samples taken from needle biopsies can be used to generate HCC organoids. With a 26% success rate, Nuciforo et al. (19) created a series of HCC organoids utilizing needle biopsies from HCC patients. Six out of ten HCC organoids were able to form tumors and multiply steadily for a long period of time after being transplanted into immunodeficient mice. It’s worth to note that the organoid culture medium plays a crucial role in the development of HCC organoids (**Figure 1**). To sustain the normal development of liver organoids during normal liver organoid cultures, specialized buffers, amino acids, and cytokines are frequently added to the basal medium (26). In the instance of HCC organoid cultures, Broutier et al. modified the medium so that it was free of R-spondin-1, Noggin, and Wnt3a but contained dexamethasone and Rho-kinase inhibitors, while Nuciforo et al. deleted forskolin, N-acetyl-L-cysteine, nicotinamide, and HGF because it was demonstrated that they had a negative effect on proliferation in HCC cells. And for its capability to promote HCC cell proliferation, FGF19 was additionally introduced.

## 3 Superior personal heterogeneity compared to 2D models

For the research of liver cancer, traditional 2D cell line models were frequently used but they still had many disadvantages. In 2016, the National Cancer Institute (NCI) announced the retirement of the NCI60 cell line, which had been used for 25 years when it was withdrawn from the drug screening system (27). It means that the era of the CDX (Cell-derived Xenograft) model has passed.

Tumor cells in the human body are found in a complex environment, and the interaction between the environment and the cells is crucial for the development, upkeep, and expression of the cells’ functional properties. The original tumor’s heterogeneity and three-dimensional structural characteristics are absent from cell line models, and *in vitro* cultivation makes the genome unstable (28). Organoid models of three-dimensional structures are better able to replicate cell-cell and cell-environment interactions than 2D models, which only retain horizontal simulations of these interactions, due to their ability to replicate the structural and functional characteristics of *in vivo* tissues. The HCC organoids can reproduce the *in vitro* histological characteristics and therapeutic response of the originating tumor (18, 19). And it is able to preserve mutational features, simulate different subtypes, and preserve a good tumorigenic potential (23). Furthermore, due to the monolayer structure of the 2D model, all the cells are exposed to the same drug concentration, which is far removed from the *in vivo* situation. The 3D structure of the HCC organoid offers the benefit of being able to replicate the targeted drug diffusion and recreate the gradient of drug concentration that is therapeutically relevant (29).

**Figure 2:**
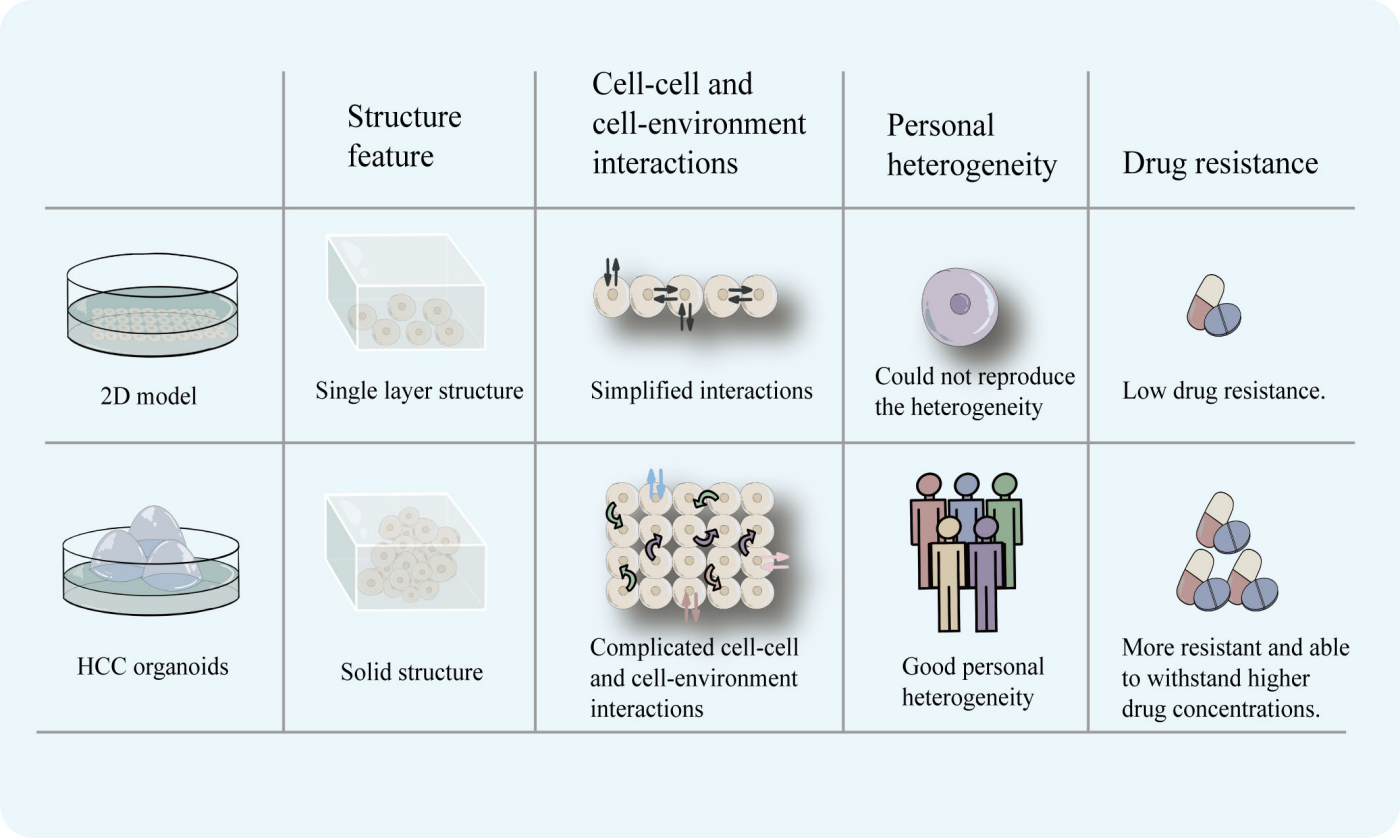
2D model has a monolayer cell structure, which can only simulate cell-cell and cell-environment communication at the planar level. HCC organoids with a three-dimensional structure can more completely simulate the complex communication between cells and cell-environment. In addition, HCC organoids can reproduce the heterogeneity of patients and is more realistically simulate the drug-resistance characteristics of tumors.

Although 2D models can represent a wide variety of disease subtypes and significant mutations, they lack the ability to predict medication response because they do not replicate interpatient variability, the complexity of the tumor microenvironment, and organ function (30, 31). In contrast, liver organoids can accurately mimic the three-dimensional structure of the organ while preserving its unique functions and genetical features (10, 18, 19). More importantly, HCC organoids are able to reproduce heterogeneity. Li et al. used 27 patient-derived organoids (PDOs) which were obtained from different regions of 5 patients with primary liver cancer (CCA or HCC) to test 129 cancer medications and discovered that there was intra-tumor and inter-patient drug response variability between PDOs. Primary tumors and matched PDOs were confirmed to exhibit similar staining for various markers (epithelial marker, the bile duct markers cytokeratin 19 and 7, the mucin marker mucicarmine, the stem cell markers LGR5 and SOX9). At the same time, after labeling, their PDOs were confirmed to exhibit a label spectrum similar to the original tumor [epithelial tissues (EPCAM), stemness (LGR5), liver origin (CK19), and hepatocyte-specific markers (AFP and HepPar1)]. Their research has shown that some molecular targeted medications, such as dasatinib and ceritinib, only had an effect on one subgroup of HCC. Also, numerous targeted medications, including sorafenib, displayed inter-patient drug response heterogeneity. Moreover, certain medications showed individual variations in drug response at different PDOs. For instance, ceritinib showed good tumor suppression in 3 PDOs, but in the other 3 PDOs, there was essentially little anticancer action (22). When administered in clinical settings, sorafenib’s efficacy varies greatly amongst patients (32), and the findings of this study are consistent with this effect. This demonstrates the HCC organoids’ ability to maintain heterogeneity and the accuracy with which the outcomes of targeted drug sensitivity testing reflect the actual world. Their study reflects intrinsic sensitivity to drugs in cells but fails to reflect complicated *in vivo* interactions. However, this research finding demonstrates the potential of using organoid models for logical medication selection. To further support the benefit of HCC organoids in preserving the heterogeneity of patient therapeutic response, more samples will be required in the future. HCC organoids may be a useful *in vitro* model for screening patient-specific targeted therapeutic therapies (**Figure 2**).

## 4 High-throughput and low-cost evaluation of sensitivity of targeted drug compare to PDX model

Except for 2D model, Patient-Derived Xenograft (PDX) is often used in the research of PLC as the standard model because of its advantages (6). PDX can preserve the genetic characteristics of the original tumor and the interaction between the tumor and stroma (33). In 2019, Blumer et al. established the PDX models from human HCC biopsies and to characterize their histologic and molecular stability during serial passaging (7). However, PDX still has limitations. The construction of PDX is time-consuming, laborious, and expensive, and it cannot simulate the interaction between tissues and the internal environment of the organism. Cultivation of a PDX usually takes several months to a year, including transplantation, *in vivo* proliferation in mice, and passaging, making it too long to meet the urgency of clinical drug use (33). And compared to PDX, the time required to construct HCC organoids is significantly shorter, making it more feasible for clinical use. In addition, HCC organoids can be cultured stably *in vitro* for long periods of time (34) and still retain their original tumor characteristics after 15 passages, which is difficult to achieve with the PDX model. Drug sensitivity testing employing HCC organoids is less time-consuming due to the easier amplification process and testing methodology.

Additionally, transplantation is an important step in the PDX construction process and immunosuppressed mice (NOD-SCID mice) are usually used. The altered immune environment will undoubtedly affect tumor function and drug response, limiting the use of PDX, especially for testing some immunotherapeutic drugs. And compared to organoid models, the success rate of constructing PDX models (35) and transplantation is lower (36). The excessive time and financial burden and the lower success rate of transplantation have limited the widespread clinical use of PDX.

**Figure 3:**
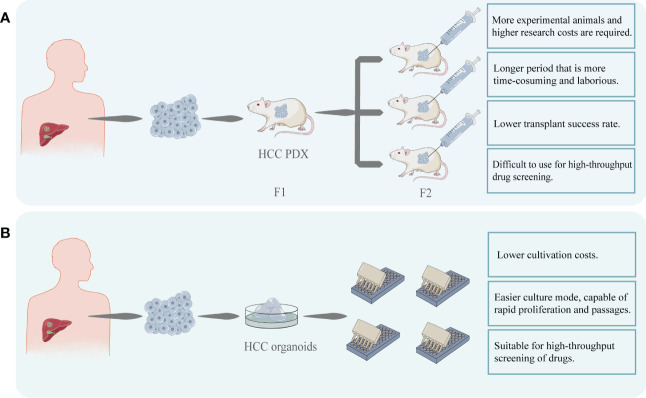
**(A)** HCC cells were obtained from patients and transplanted into immunodeficient mice to construct HCC PDX. After successful transplantation, the mice can be cultured for passaging and used for targeted drug testing. PDX models are costly and time-consuming to construct and difficult to perform rapid, large-scale drug screening. **(B)** HCC organoids can be created using patient-derived HCC tissues. This model can be easily proliferated and passaged and is stable while retaining heterogeneity. In comparison to PDX, its modeling and passaging are easier and quicker, making it more suited for high-throughput and low-cost drug screening.

The monetary investment in the development of a drug is undoubtedly one of the concerns of the investigator. A drug that performs poorly in the clinical phase or suffers a delisting can result in significant financial losses. HCC organoid models have the advantage of reducing the risk of drug trial failures or delisting, helping to reduce the associated financial losses (37). HCC organoid production is less complex than PDX, takes less time, and has superior amplification and passaging characteristics. These make it possible to use HCC organoids for high-throughput drug screening at a relatively short time and fast test pace compare to PDX (36)(**Figure 3**). Therefore, these factors will help to reduce the cost of targeted drugs sensitivity evaluation.

## 5 More reliable efficacy of targeted drugs sensitivity screening with HCC organoids

It has been demonstrated that liver organoids can successfully mimic the functions of the liver *in vivo* (10) and can proliferate for an extended period of time (18, 38). And one of the most prominent advantages of HCC organoids models for drug sensitivity testing is their good reproducibility and fidelity. This allows the HCC organoid to be used as a good *in vitro* model for sensitivity testing to molecular targeted drugs, such as sorafenib (19). Compared to other models, organoid models can reproduce disease features such as mutational characteristics, pathological changes and treatment response (18, 19) and are more realistic in terms of drug response.

As previously indicated, a variety of factors, like oversimplified cellular interactions and monolayer structures that affect drug penetration, have an impact on how realistically a medication is sensitive in 2D models. On the other hand, the structural benefits of 3D HCC organoid models enable more precise therapeutic response prediction (39). It is worth noting that, Song et al. used PDOs to test the sensitivity of sorafenib and found that 3D structures of the organoid showed higher drug sensitivity compared to the 2D model. This is not routinely understood – the sensitivity is reduced because the 3D structure hinders drug diffusion (29). This interesting finding confirms the authenticity of the drug response of the organoid from the side. Drug response in tumors cannot be predicted by direct extrapolation from the results of 2D model experiments, and 3D structural features are not the only factors that affect drug transport and diffusion in tissue structures.

At the same time, at the level of gene expression, the expression of genes associated with mediating tumor drug resistance is higher in the 3D HCC organoid than in the 2D model (40). Higher expression of drug-resistance genes leads to stronger treatment resistance, which means that drug testing using HCC organoids is subject to higher test drug concentrations. And in comparison to the 2D model at the same drug concentration, the 3D organoid model is more viable and displays a higher level of targeted drug resistance (41). And Sun et al. confirmed that the IC_50_ obtained in the sensitivity testing of molecular targeted drugs using the 3D organoid model was higher than the one in 2D model and closer to the real effective blood concentration in humans (40). This undoubtedly confirms the realism of the simulation of resistance of HCC organoids to tumor treatment and makes targeted drug sensitivity testing more reliable. Routinely prescribed targeted medications like sorafenib and levatinib have issues with resistance when used to treat advanced HCC (32, 42, 43). Although the medications can increase a patient’s chance of survival by roughly 3 months, resistance frequently arises within 6 months, which has an impact on the clinical outcome (32, 43). The therapeutic outcome is significantly impacted by the variation of patient medication responses. Personalized medical treatment for different patients is undoubtedly necessary. HCC organoids are able to express drug resistance while preserving the heterogeneity of patient tumor characteristics, thus demonstrating the heterogeneity of sensitivity and resistance to targeted drugs across patients. This is unquestionably advantageous and offers the potential to serve as a good model for directing targeted drug use and offering patients individualized medical care. The analysis of gene expression in drug-resistant HCC organoids can also reveal the relationship between gene up- or down-regulation and the evolution of drug resistance (44, 45). And HCC organoid resistance can be instilled, as was the case when Tong et al. built up organoid resistance to sorafenib by gradually increasing the drug concentration (46). And despite inducing resistance, the organoid can still maintain inter-patient heterogeneity and be better for determining the drug’s direct impact on tumor cells (36). Thus, HCC organoid model is more feasible.

Combinations of drugs are commonly used in clinical settings and are often used to enhance the therapeutic effect. HCC organoids also have advantages in testing the combination of targeted agents. By combining existing therapeutic options with other drugs, suitable drugs can be screened to enhance the sensitivity to certain targeted drugs and reduce drug resistance issues. New therapeutic options can be discovered using HCC organoids. For example, It has been demonstrated that CD44 positivity and Hedgedog signaling play a crucial part in the development of chemoresistance in some malignancies (47). Wang et al., after that, confirmed the relationship between Hedgedog signaling and CD44 positivity and sorafenib resistance using HCC PDOs. They showed that sorafenib was much less effective against CD44-positive PDOs and that the Hedgehog signaling inhibitor GANT61 worked in concert with sorafenib (20). Similarly, it was discovered that sorafenib-resistant individuals had considerably higher expression levels of Src homology 2 domain-containing phosphatase 2 (SHP2). By obstructing this signaling pathway, SHP099 serves as a sorafenib sensitizer, eliminating sorafenib resistance in HCC organoid (48).

**Figure 4:**
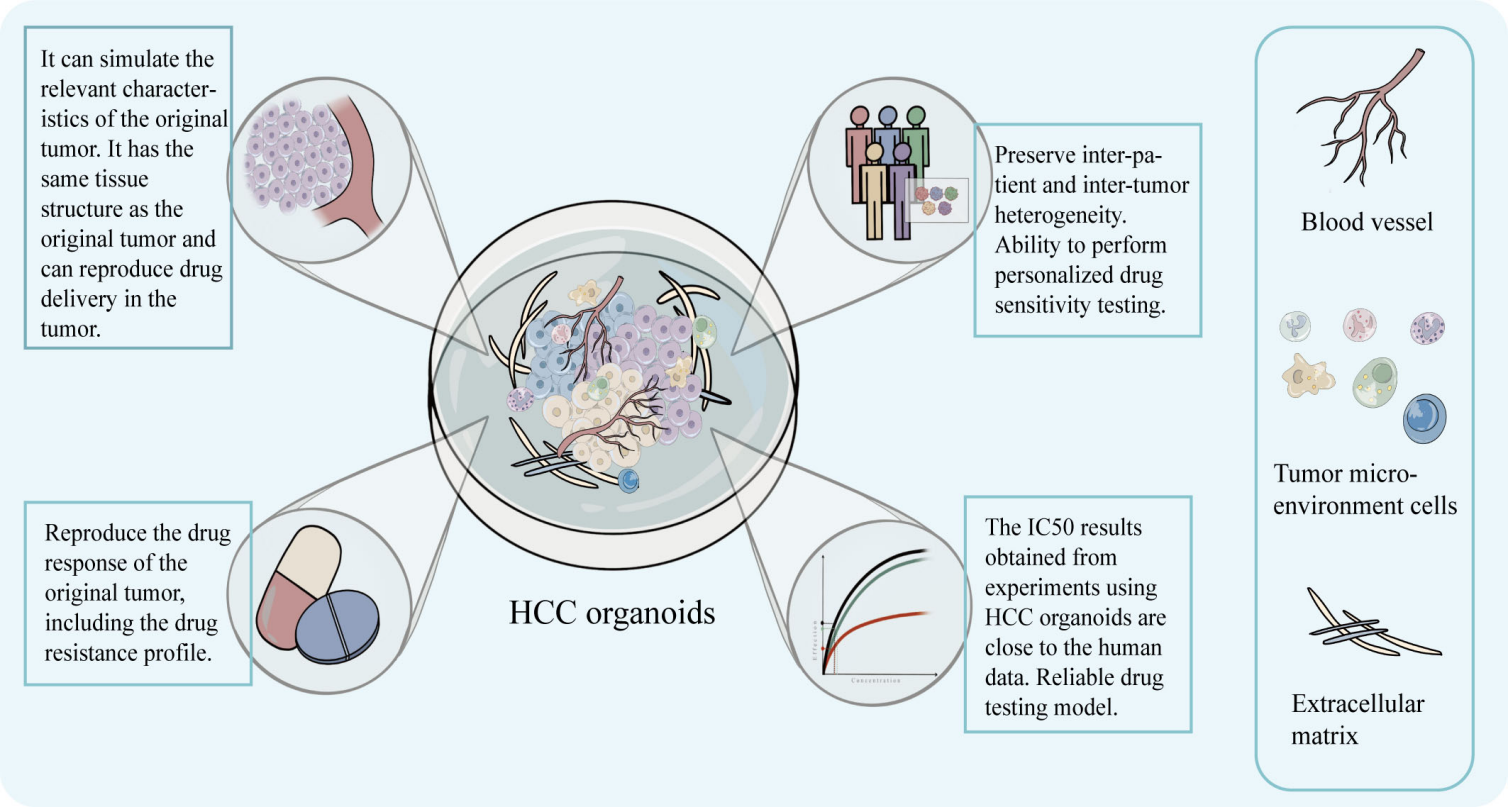
The model can preserve the structural characteristics of the original tumor, has advantages in simulating the drug concentration layer related to the treatment and can obtain IC50 that is closer to the real data of the human body. It can preserve the intra-tumor and inter-patient heterogeneity, drug response, and drug resistance characteristics, and help realize personalized targeted drug susceptibility testing.

Additionally, a major worry for researchers is the concordance of results from *in vitro* models with those from clinical investigations. Studies have shown that the results of sensitivity testing for specific molecular targeting drugs, like sorafenib and regorafenib, were in line with clinical findings when done using HCC organoid (36). For instance, Tong et al. discovered that sorafenib and ANXA3 mAb combined treatment reversed the growth of HCC organoid and that suppression of ANXA3 increased the organoid’s sensitivity to sorafenib and regorafenib. The results of this experiment were validated in an *in vivo* trial (46). The HCC PDOs created by Dong et al. demonstrated individual-specific sensitivity to a number of medications, including sorafenib, and can be supported for use in clinical settings (49). The authenticity of the organoid was verified by the consistency of the drug sensitivity test results with the clinical response (**Figure 4**).

## 6 The disadvantage and perspective of HCC organoids for targeted drugs sensitivity screening

**Figure 5:**
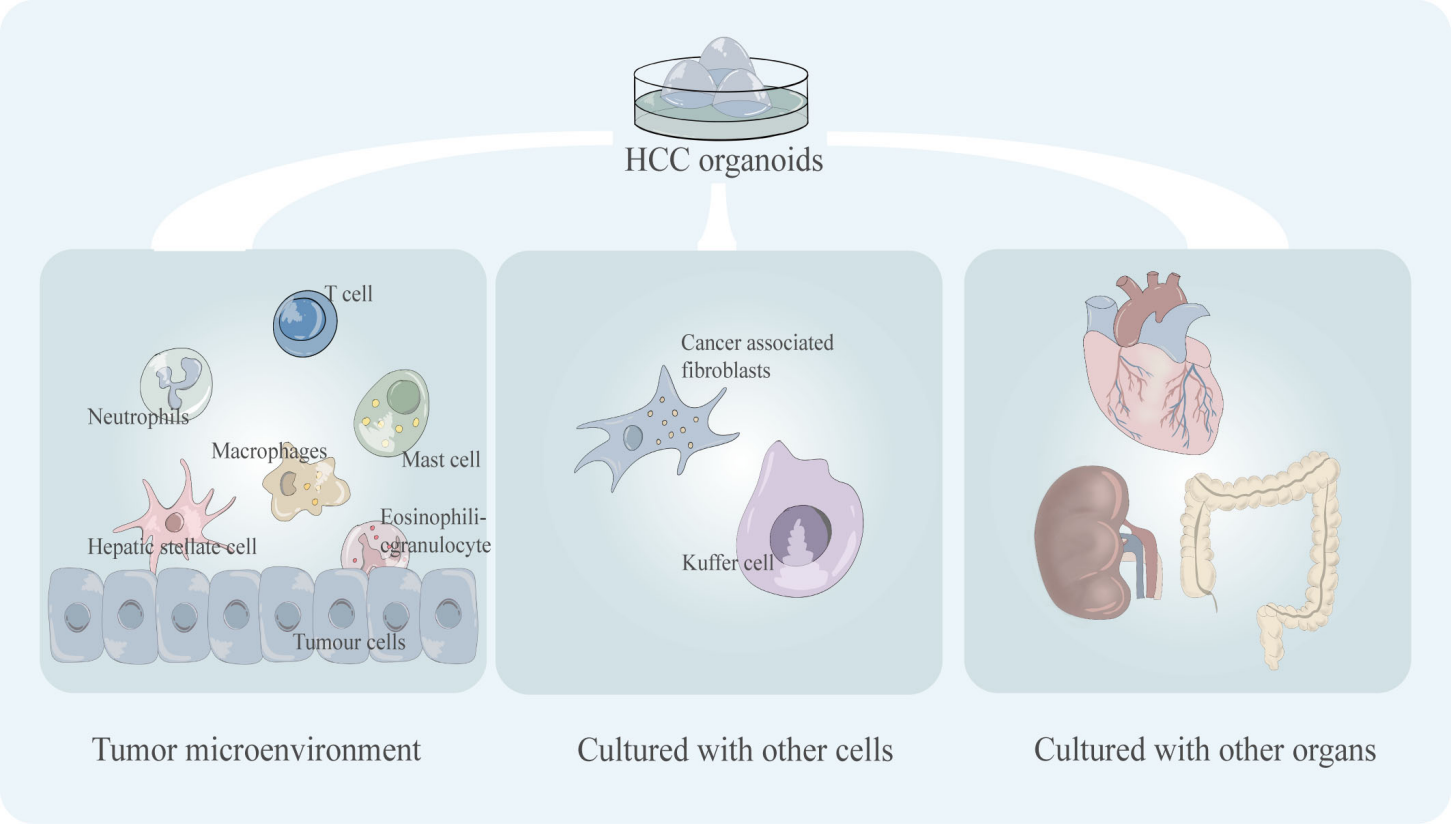
Key shortcomings of existing HCC organoid models including the incomplete tumor microenvironment (tumor immune microenvironment) and the lack of co-culture mode, including co-culture with multiple cells and co-culture with multiple organs.

As an evolving model, the organoids is limited in its application due to some disadvantages (**Figure 5**). One of the widely discussed drawbacks is the lack of a relatively realistic simulation of the tumor microenvironment (TME) *in vivo.*

The TME contains a variety of different cell types, such as tumor-infiltrating lymphocytes which are an important part of the TME, and the variability in cell composition type and phenotype which can lead to significant differences in treatment outcomes (50). Some immunotherapeutic drugs target T cells to activate the tumor-killing ability of T cells, such as PD-1 inhibitors. For example, nivolumab, pembrolizumab, and nivolumab plus ipilimumab are immune checkpoint inhibitors approved by the FDA for the treatment of HCC (51). The lack of the immune microenvironment of the organoid leads to difficulties in the simulation of tumor drug response in immunotherapy, which will undoubtedly limit its application in the testing of such drugs. Numerous cell types, including Carcinoma Associated Fibroblasts (CAFs), Hepatic Stellate Cells (HSCs), Tumor-Associated Macrophages (TAMs), and Immune and Inflammatory Cells, are present in the TME of HCC and have a significant impact on the effectiveness of targeted drug therapy for HCC (52). For instance, sorafenib and regorafenib, two extensively used clinical target medicines, conferred resistance to co-culture models of HCC harboring CAFs (53). Compared to simple models, multicellular co-cultured HCC organoids are more responsive to medicines like sorafenib (29). Thus, co-culture models of organoids with various cell types and a wider variety of immune cell types still need to be developed.

The culture environment of organoid models is relatively simple, and artificial reconstruction of relatively realistic *in vivo* TME has always been a great challenge for tumor research. The Calvin J. Kuo research group of Stanford University in the United States cultured PDOs by the air-liquid interface (ALI) to reproduce the patient’s TME. This research result was published in the journal *Cell* in 2018. Their model, which was validated at the gene level, successfully conserved the fibrous matrix and immune cell components in the original tumor tissue. They also demonstrated that PDOs conserved the T, B, and NK cells that were present in the initial tumor (54). Their experimental results provide a feasible solution for reconstructing TME *in vitro*. ALI-PDOs may help the reappearance of TME in organoids and facilitate tumor research. In addition, co-culture of cells of a specific type within TME with tumor organoids can also help partially characterize TME. This has been successful in some tumor organoids (53, 55, 56).

Another drawback of organoids is their inability to accurately simulate the intricate nature of multi-organ interactions *in vivo.* The main physiological processes and pharmacological responses of particular organs can be replicated in organoids, but the inability to mimic *in vivo* interactions and drug metabolism impairs the accuracy of drug sensitivity testing in predicting clinical responses to drug use. In the body, each organ maintains its own independence while maintaining communication with other organs, and how to mimic this communication between tissues and organs *in vitro* has always been a challenge. Therefore, linking several organs in series in a common medium and replicating the communication between many organs in a common circulation can help comprehend the interaction between organs and the systematic impacts of the environment on multiple organs. Replicating the complexity of interactions in humans by stringing together organoids allows high-fidelity simulation of physiological, pharmacokinetic, and pharmacodynamic features to obtain predictions that are more consistent with clinical studies. The co-culture model with multiple organs/organoids in series may be a promising model. Researchers have created a two-organ co-culture model comprising neurospheres and a liver organoid using organ-on-a-chip technology. Co-cultured multiple organs were found to have a greater toxicity response and increased sensitivity in later toxicity trials (57). The possible reason for this is that apoptosis in one organ induced increased sensitivity in the other organ (57). It is easy to see that organ interactions have important effects on drug responses (58).

**Figure 6:**
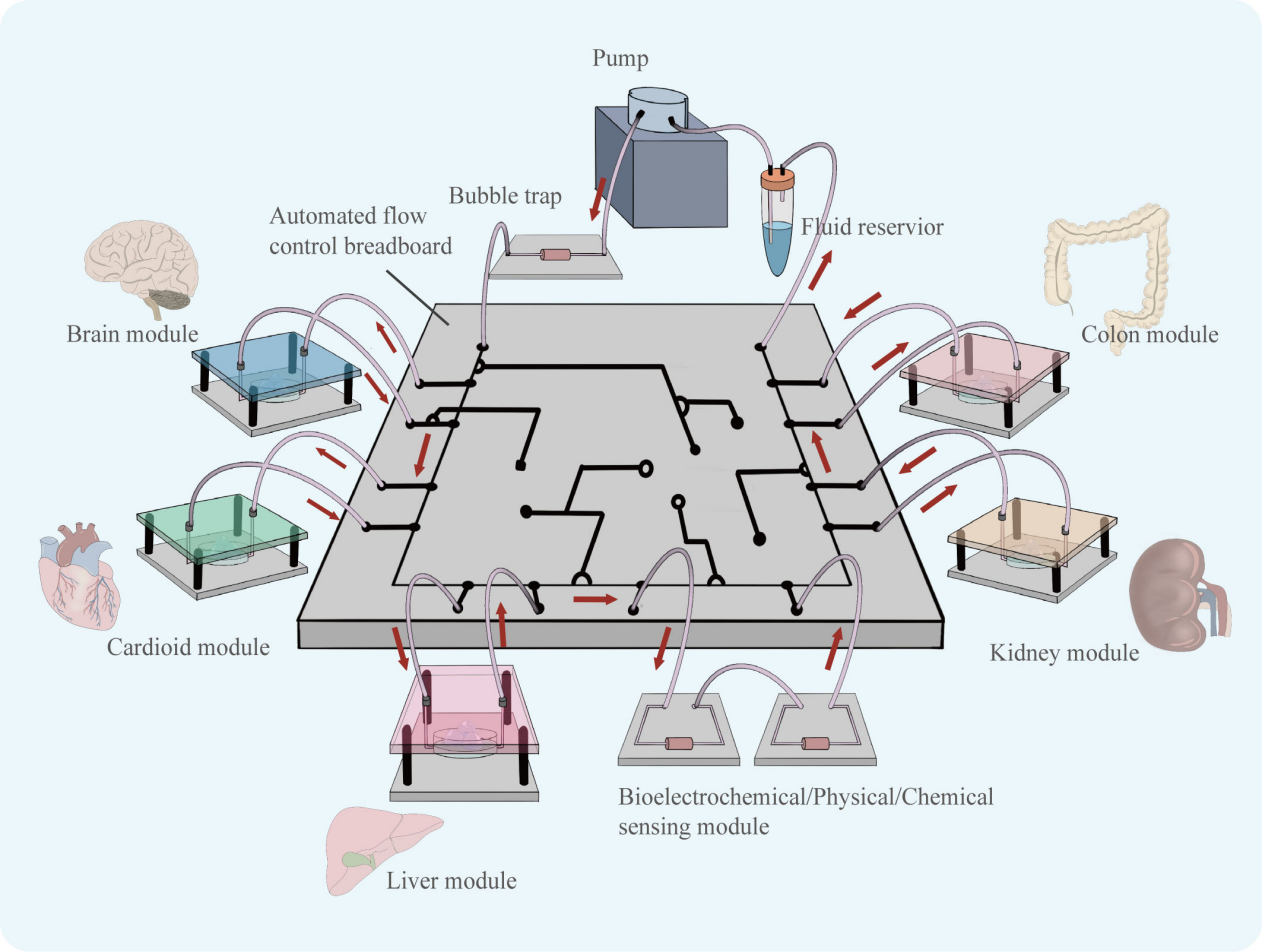
Organoids of various organs can be derived using similar culture patterns. Assembling cultured organoid models in a chip controlled by a microfluidic system, the flow of fluid can simulate the flow of material between organs. The organoids-on-a-chip is able to simulate drug therapy in a multi-organ co-culture mode.

More, the combination of organoid technology and organ-on-a-chip technology can construct organoids-on-a-chips as a new model may be a good model that can well characterize the complexity of tumors’ environment *in vivo* and greatly facilitate the development of anti-tumor drugs in the future. In 2019, a review published in the journal Science first introduced the concept of organoids-on-a-chip (59). Organoids-on-a-chip is also considered to be one of the most cutting-edge directions in organ-on-a-chip development. Organ-on-a-chip builds three-dimensional human organ physiological microsystems on a chip using microfluidic technology to control fluid flow, combining intercellular interactions, matrix properties, as well as biochemical characteristics and biomechanical properties, which can precisely manipulate the microenvironment of organs and control the volume of microtissue organs in a very small range. It is a reliable model for anticipating how people will react to drugs (60). The use of microfluidics can improve the ability of organoids to simulate tumors *in vitro*, and by precisely controlling physical and chemical gradients, the state of cells can be modulated to better simulate drug responses (61). There is still much room for the development of co-culture organoid models (**Figure 6**). Among the targeted drugs for liver cancer treatment, many play an anti-tumor role by inhibiting liver cancer angiogenesis, such as sorafenib (62) and apatinib (63).The co-culture of HCC organoids and vasculature is particularly crucial for the sensitivity screening of such targeted medications, and the development of this model will significantly advance research on anti-angiogenic targeted drugs. Shao et al. Using new scaffolds and microfluidic systems to construct a multi-layer assembled co-culture chip of hepatocytes-fibroblasts-vascular endothelial cells (64). The findings of this study offer suggestions for the further construction of organoids-on-a-chips. In addition, co-culture of HCC organoids with organs such as intestine and kidney on a chip may be able to characterize the pharmacokinetic landscape of HCC-targeted medications and further enrich the research results of targeted drugs.

Although HCC organoids have the potential to be used in the clinic, especially in the area of personalized medicine, the process of therapeutic transformation is hampered by their poor initiation success rate. It is necessary to pay attention to the issue of how to increase the initiation success rate of HCC organoids and make it feasible to benefit more patients. It is simple to believe that the quality of the obtained samples and the handling of the samples affect the initiation of organoids. Therefore, care needs to be taken when obtaining samples, and as many living cells as possible should be preserved to avoid the collection of necrotic tissue. In addition, a standard sample collection and transport procedure is necessary in order to protect the sample’s vitality and keep its ischemia duration to a minimum (65). A high initiation rate suggests that more patients may gain from the treatment and an effective and efficient initiate procedure needs to be enhanced. Additionally, only low- and moderately differentiated tumors were able to successfully grow HCC organoids (66, 67), indicating the necessity of early tumor diagnosis and treatment intervention. Furthermore, the lack of a large-scale standardized procedure for organoid construction, the uncertainty of construction results (31), and the lack of realistic mimic *in vivo* tumor environment leading to the failure of testing HCC monoclonal antibodies remain to be addressed (68). And, additional clinical samples must be used to confirm the accuracy of the results of the sensitivity testing of targeted medications utilizing the HCC organoid (19).

The HCC organoids still have a lot of space for advancement, and combining them with some existing methods may make them even more feasible for targeted drug sensitivity testing. For example, genetic testing technology and the CRISPR-Cas9 system. The genetic technology has played a key role in the development of precision medicine, especially in the use of targeted drugs and immune checkpoint inhibitors (69). Genetic testing technology combined with organoid models can further promote the development of precision medicine. By using gene sequencing to screen for therapeutically sensitive drug targets and then using organoids to verify target sensitivity, the accuracy of drug administration can be improved, leading to better clinical outcomes. Additionally, CRISPR-Cas9 is a molecular biology technique that allows for the editing of genes in cells and organisms. The technology can help understand tumorigenesis and can assist in the discovery of new drugs (12, 70). Combined gene editing techniques allow the introduction of specific mutations in HCC organoids. Drug sensitivity testing is performed for different mutation types and the results obtained can be composed into a database that may help in the adjustment of treatment strategies for patients carrying the relevant mutated genes (71). It is also useful to understand the relationship between different mutated genes and the development of drug resistance.

## 7 Conclusion

As a promising *in vitro* model for targeted drug susceptibility testing, the HCC organoids have the advantage of reproducibility and fidelity, reproducing the histological, genetic, and functional characteristics of the original tumor, maintaining good heterogeneity and tumorigenic potential, as well as long-term stable proliferation *in vitro* and maintaining relevant tumor characteristics after multiple passages. HCC organoids are able to reproduce patient heterogeneity on the basis of simulated tumor characteristics, including response to targeted drug therapy and targeted drug resistance. It can also be used to perform high-throughput drug screening and enables low-cost, rapid, and realistic evaluation of patient sensitivity to targeted drugs. They may be valuable in many ways including helping to address therapeutic resistance issues, screening for potentially effective drugs and assisting in personalized medicine. In conclusion, HCC organoids have shown to be advantageous as models for targeted drug sensitivity testing.

But as a still evolving *in vitro* model, HCC organoids still have drawbacks, such as incomplete microenvironmental simulations the lack of the simulation of the intricate nature of multi-organ interactions *in vivo*, and the scarcity of samples, which makes large-scale applications difficult. In addition, the reliability of HCC organoids for targeted drug testing still needs to be confirmed with more samples. The construction of co-culture models and organoids-on-a-chips will be a promising development that will help solve existing problems and lead to significant progression.

## Author contributions

CX and AG completed the writing of the first draft and the drawings of the manuscript. MK and BC were responsible for revising and proofreading articles. BC and ZX were in charge of supervision. XY, LC, JH, YW, HS, JC, and JL were responsible for data collection and collation. FY, PW, FY, and FT were responsible for software management. All authors contributed to the manuscript revision, read, and approved the submitted version.
